# Critical assessment of furrow openers and operational parameters for optimum performance under conservation tillage

**DOI:** 10.1038/s41598-024-70569-2

**Published:** 2024-09-09

**Authors:** B. S. Madhusudan, H. L. Kushwaha, Adarsh Kumar, Roaf Ahmad Parray, Sidhartha Sekhar Swain, Manojit Chowdhury, Ramineni Harsha Nag, K. R. Asha, Sunil Kumar Rathod, Pradeep Kumar, Rohit Anand, Nadhir Al-Ansari, Ahmed Z. Dewidar, Mohamed A. Mattar

**Affiliations:** 1 Department of Agricultural Engineering, REVA University, Bengaluru, 560064 India; 2 Division of Agricultural Engineering, ICAR-Indian Agricultural Research Institute, New Delhi, 110012 India; 3 Department of Civil, Environmental, and Natural Resources Engineering, Lulea University of Technology, 97187 Luleå, Sweden; 4 Prince Sultan Bin Abdulaziz International Prize for Water Chair, Prince Sultan Institute for Environmental, Water and Desert Research, King Saud University, Riyadh, 11451 Saudi Arabia; 5 Department of Agricultural Engineering, College of Food and Agriculture Sciences, King Saud University, Riyadh, 11451 Saudi Arabia; 6 Department of Farm Machinery and Power Engineering, University of Agricultural Sciences, Raichur, 584104 India

**Keywords:** Conservation agriculture, Sustainable agriculture, Draft requirements, Residue management, Straw cutting efficiency, Working speed, Agroecology, Mechanical engineering

## Abstract

Conservation Agriculture (CA) is an innovative approach that promotes sustainable farming while enhancing soil health. However, residue management challenges often hinder its adoption, causing farmers to burn crop leftovers in fields. This study aimed to evaluate the effectiveness of various furrow openers under simulated soil bin conditions. Three types of furrow openers were examined: single disk (SD), Inverted T-type furrow opener with a plain rolling coulter (ITRC), and double disc (DD) furrow opener. Tests were conducted at different forward speeds (1.5, 2, and 2.5 km h^−1^) and with three straw densities (1, 2, and 3 t ha^−1^) at a consistent working depth of 5 cm. Draft measurements were obtained using load cells connected to an Arduino-based data-logging system. Results indicated that draft requirements increased with forward speed and straw density, while straw-cutting efficiency decreased with these factors. Average draft values for SD, ITRC, and DD were 290.3 N, 420 N, and 368.5 N, respectively, and straw-cutting efficiencies were 53.62%, 59.47%, and 74.89%, respectively. The DD furrow opener showed the highest straw-cutting efficiency (81.36%) at a working speed of 1.5 km h^−1^ and a straw density of 1 t ha^−1^, demonstrating optimal performance compared to other furrow openers.

## Introduction

Every year, in India, the rice–wheat cropping system produces approximately 500 million metric tons of crop residue^1,2^. Inadequately processed crop residues can obstruct subsequent farm operations, block furrowing, hinder seed placement, reduce sowing quality, delay seed germination, and ultimately reduce yield^3,4^. To address these issues, farmers often resort to on-field crop burning as an alternative, which leads to environmental pollution and a decline in soil nutrition level^5,6^.

Conservation tillage (CT) has emerged as a viable solution to enhance the efficient utilization of crop residues^7–10^. However, the mechanical disruption caused by crop residues during sowing operations poses a major challenge to the successful implementation of CT^11–13^. CT uses various type of furrow openers, including hoe, disc, sweep, and chisel type, to open furrow grooves and manage crop residues^14–16^. Furrow openers play a crucial role in crop residue cutting and forming furrow grooves, which are essential for successful conservation practices. Each type of furrow opener has unique characteristics, with variations in draft force requirements and straw-cutting capabilities, making the selection of a suitable furrow opener crucial for effective crop residue management^17,18^.

Disc type furrow openers and coulters are preferred over hoe type furrow openers for their ability to minimize soil disturbance and aggressively cut crop residues^19–21^. In 2001, Chaudhury^22^, introduced an inverted-T opener, which resulted in higher germination percentages compared to other furrow openers. Fallahi^23^, reported that integrating rolling coulter attachments into planters can enhance seeding performance in CT systems. Among the available furrow openers, the double disc furrow opener is recognized as the most effective.

Several factors significantly influence the performance of furrow openers used in CT, including residue cutting efficiency, draft requirement, and vertical forces. These factors include operational speed, straw density, type of furrow opener, and the degree of soil compaction^24–26^. In a soil bin study carried out by Kushwaha 11 in 1986, they employed smooth disc coulters with diameters of 360 mm, 460 mm, and 600 mm. The experiment involved varying the operating depth between 50 and 70 mm, as well as adjusting the amount of crop residue in the range of 1000–5000 kg ha^−1^. The study found an inverse correlation between straw density and the amount of residue cut, while the amount of residue cut increased with working depth and soil strength, as indicated by the cone index. In 2006, Sahu et al.^26^, reported that the draft required for furrow openers is directly proportional to soil compaction, operating depth, and speed. Ahmad et al.^14^, evaluated the impact of double disk furrow openers on draft and straw cutting ability in no-till residue conditions. The researchers determined that both operational speed and straw density greatly influence the draft and the degree of straw cutting. In no-till soil conditions, Xu et al.^27^, found that the tillage performance of fluted coulters is more influenced by the working speed and straw density rather than the coulter geometry. In 2019, McLaughlin et al.^28^, reported that operational speed influences both the force requirement and residue-cutting ability of the furrow opener. Increasing the speed reduces uniformity in soil disturbance and straw-cutting performance. Sawant et al.^29^, assessed the effect of forward speed on the force requirement of tine-shaped furrow openers in simulated conditions. They found that the force increases as the forward speed increases and suggested that a forward speed of 1.76 km h^−1^ is the optimum condition for the sowing operation.

Even though various researchers have previously assessed the effectiveness of different furrow openers in terms of soil disturbance, straw-cutting efficiency, and draft force under simulated soil bin conditions^14,30^, their specific implications for the Indo-Gangetic rice–wheat cropping system remain uncertain and require further research. To address these issues, this experimental study was undertaken to investigate the impact of different forward speeds and straw densities on the draft requirements and residue-cutting ability of three distinct furrow openers under controlled soil bin conditions.

## Materials and methods

**Fig. 1:**
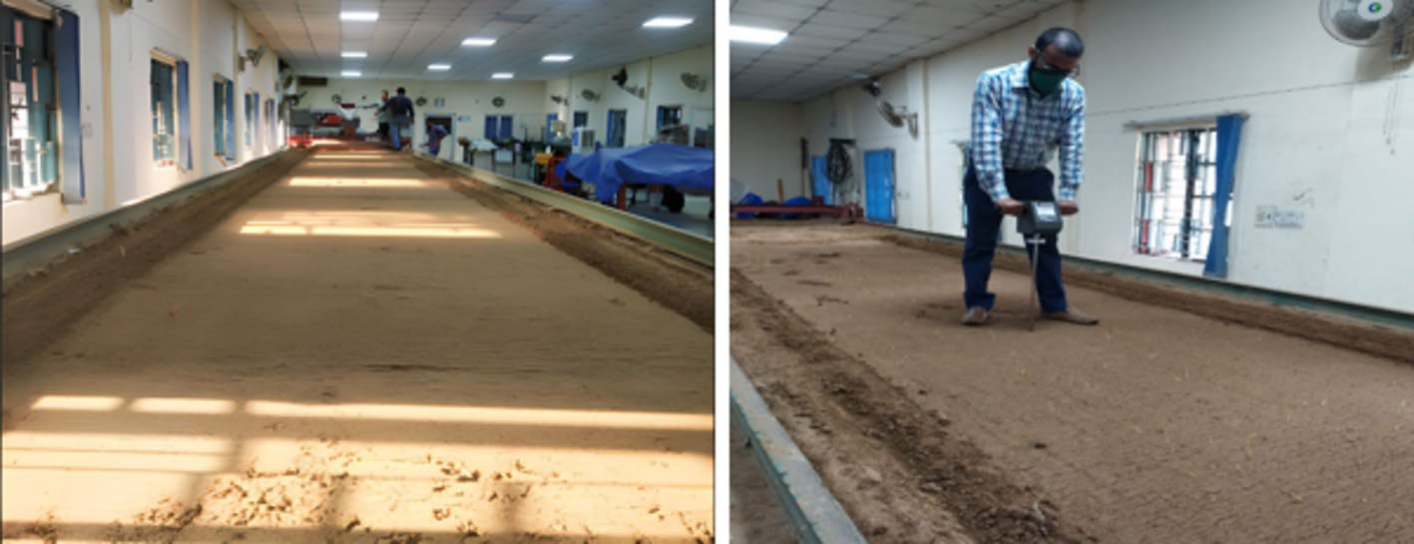
Soil bin preparation for the study of different furrow openers.

During the period of 2022–23, a series of experiments were conducted at the Department of Agricultural Engineering in IARI, New Delhi, India. These experiments took place in a soil bin with precise dimensions of 25 m in length, 1.8 m in width, and one meter in depth. The soil used in the bin was loamy clay with a composition of 80% sand, 10% clay, and 10% silt, as shown in Fig. 1. Prior to the experimental trials, the moisture content, bulk density and penetration resistance of the soil in actual field conditions were recorded, found to be 12% (db), 1.5 g cc^−1^ and 1.45 MPa, respectively, to simulate the soil condition in the soil bin to actual field conditions. To prepare for the experiments, the soil was tilled beyond the working depth using a tiller. In order to replicate real field conditions, the soil bin was adjusted to simulate the physical characteristics of the soil, including moisture, bulk density, and cone index. Subsequently, the soil bin bed was prepared by leveling and compacting it with a cylindrical roller made of iron, 30 cm in diameter, 1.8 m long, and weighing 1015 kg. Prior to conducting the test trails, three soil samples were randomly collected to determine the initial soil moisture content and bulk density. These samples were later dried in a hot air oven at 105 °C for 24 h and reweighed to calculate the soil moisture content (on a dry basis) and dry bulk density, following the gravimetric technique as described by Reynolds^31,32^.

In order to maintain uniform and consistent compaction throughout the study, a cone penetrometer (Gilson, HM-559A) was employed. This penetrometer featured a 60° cone angle, a 1.5 cm^2^ area, and a rated gauge pressure range of 0–70 kg cm^−2^, allowing for precise and accurate measurements of soil resistance. Three measurements of soil penetration resistance were taken prior to the test to ensure uniformity of compaction. The soil-tool trolley was operated with the objective of attaining standardized conditions for the experiment, which included achieving a consistent bulk density of 1.5 g cc^−1^ and a soil penetration resistance of 1.45 MPa.

To ensure consistent and repeatable conditions, the process of simulating the soil bin conditions with real field conditions was repeated after each experimental trial. This involved re-tilling the soil to the appropriate depth, adjusting the soil’s physical properties to match the initial conditions, and re-leveling and compacting the soil bed using the same cylindrical roller. By repeating these steps, uniform soil conditions were maintained throughout the study, ensuring the reliability and reproducibility of experimental results.

### Description of furrow openers selected for the study

The study examined three types of furrow openers: the single disk (SD), the inverted T-type furrow opener with plain rolling coulter (ITRC), and the double disk (DD) furrow opener. The SD furrow opener had a high carbon steel disc with a diameter of 350 mm and was tilted at an angle of 12° in the direction of travel, aiming to minimize soil disturbance while efficiently cutting residues and opening a narrow slit. The ITRC furrow opener consisted of a single 350 mm coulter that effectively cut crop residues and made a fine furrow groove in the soil.

**Fig. 2:**
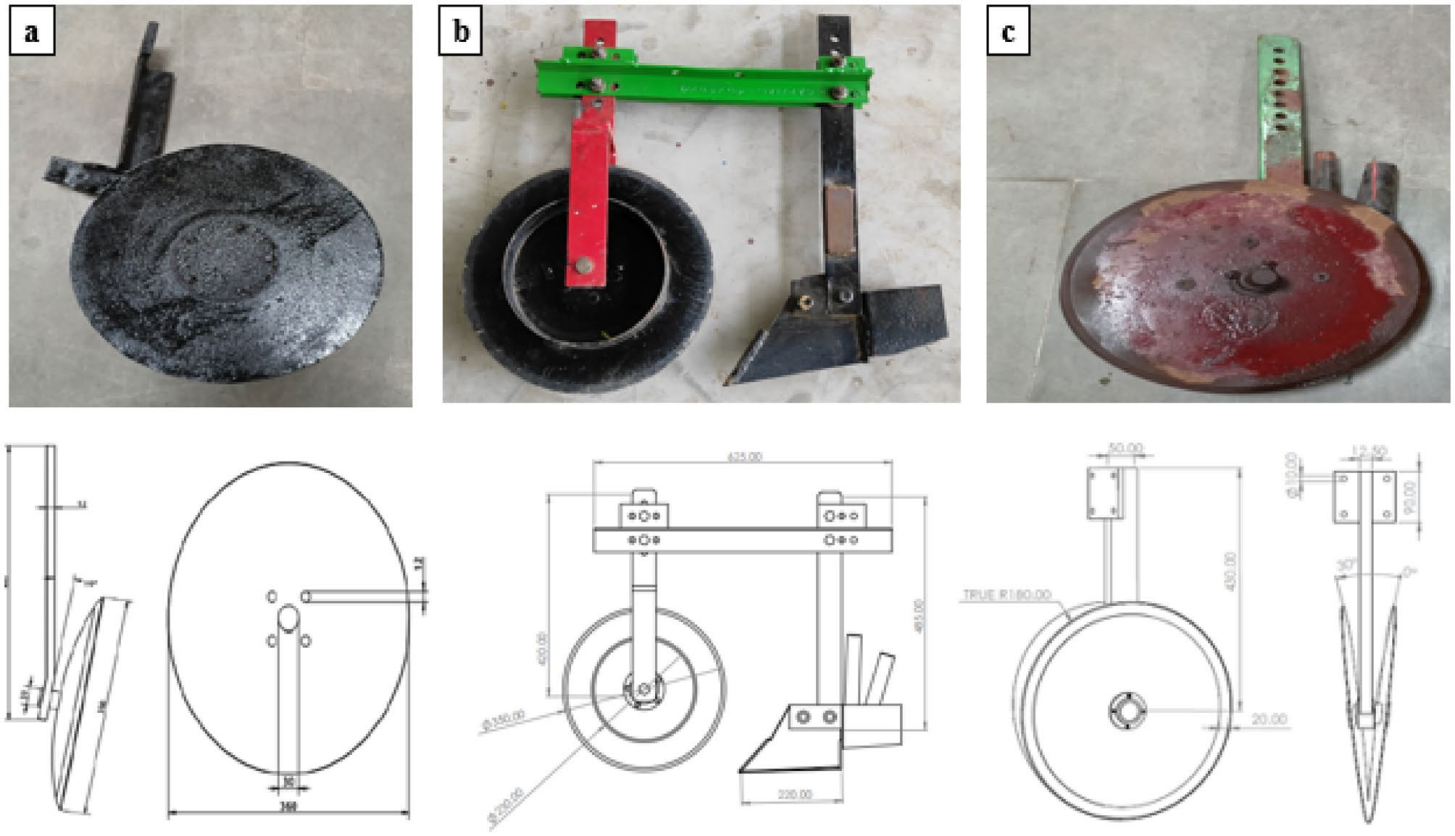
Various furrow openers employed for experimental purposes, (**a**) SD furrow opener, (**b**) ITRC furrow opener, (**c**) DD furrow opener.

Subsequently, an inverted T-type furrow opener was employed to widen the furrow groove by pushing the soil outward. In contrast, the DD furrow opener consisted of two simple rolling discs arranged to cut and move the soil outwards and downward, resulting in a V-shaped furrow groove in the soil (Fig. 2).

### Experimental procedure

**Fig. 3:**
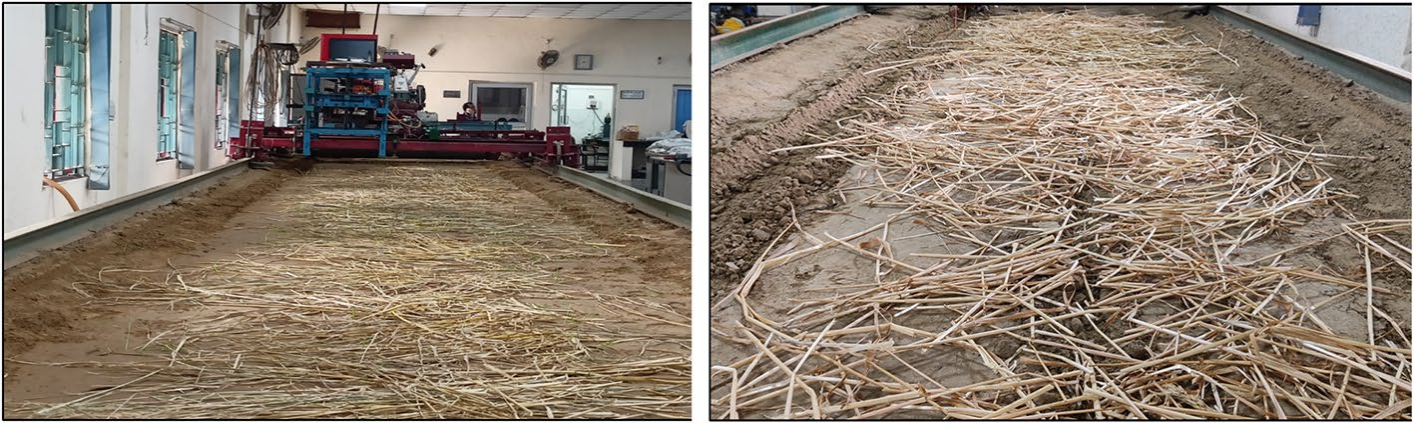
Soil bin with straw cover.

**Fig. 4:**
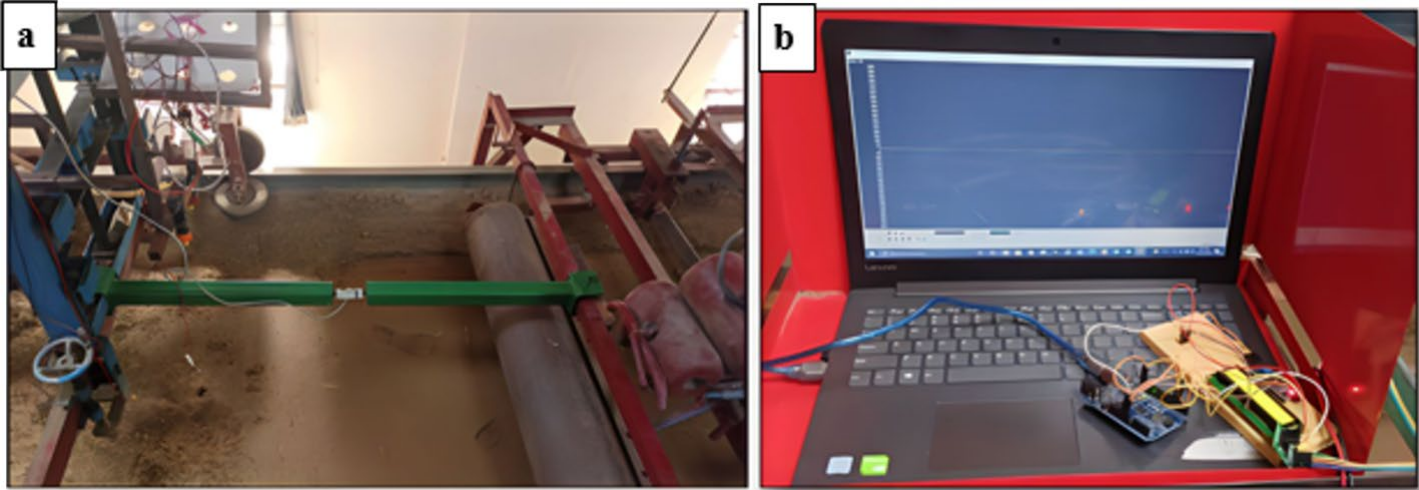
(**a**) Load cell setup. (**b**) Data acquisition window for draft measurement.

The rice crop in the Indo-Gangetic Plain is mainly harvested using combine harvesters. A field study was conducted before the experimental trials in the soil bin to measure the amount of rice straw retained in a combine-harvested paddy field. The straw retained on the field after the passage of the combine harvester was measured randomly to calculate the density of straw retention. The density of rice straw retention was found to be in the range of 1–3 t ha^−1^, consistent with the findings of references^33–35^. To investigate the draft force requirements and residue-cutting ability of furrow openers, freshly harvested rice straw with a moisture content of 18% (Wb) was uniformly spread on the soil bin surface at densities of 1, 2, and 3 t ha^−1^ (Fig. 3). The furrow openers were fixed on a frame with a depth adjustment mechanism, and an S-Type load cell (GUANG CE, YZC-516C) with a 1960 N rated capacity was attached between the frames to measure the draft (Fig. 4a). As reported by studies^36–38^, the optimum forward speed for effective sowing in Indian agricultural fields lies between 1.2 and 2.8 km h^−1^. Therefore, operating speeds of 1.5, 2, and 2.5 km h^−1^ were selected as experimental variables for this study. A trolley capable of moving freely on the soil bin at these speeds was used for the experiments. As the furrow opener moved, data on draft forces were recorded through a serial oscilloscope software with an Arduino interface (Fig. 4b).

Following the passage of the furrow opener, the straw was separated into two distinct parts: cut and uncut straw. The weight of the uncut straw was then measured by using digital scale, and Eq. (1) was applied to calculate the percentage of straw that was cut by the furrow opener.

1 $${\text{Straw cutting efficiency }}\left( \% \right) \, = { 1}00 \, {-}{\text{ Percentage of uncut straw}}$$

$$Percent \;of\; uncut \;straw = \frac{Weight \;of\; uncut\; straw, \;gm}{{Weigt \;of\; total \;straw\; spread, \;gm}}100$$

The parameters utilized to investigate the efficiency of furrow openers, specifically in terms of draft and straw-cutting efficiency, were analyzed using a response surface methodology (RSM) design known as central composite design (CCD). The CCD method enabled optimal parameter optimization with a minimal number of experiments and facilitated the examination of parameter interactions^39,40^. Table 1, in the study presents the experimental ranges of the factors used, while Table 2, illustrates how these ranges correspond to the levels in the experimental design.

**Table 1:** Characterizing experimental factors and corresponding coded levels in CCD.

Factors	Units		Level of independent variables				
			− α (− 1)	Lowest (− 1)	Middle (0)	Highest (+ 1)	+ α (+ 1)
A: Furrow openers	Num		SD	SD	ITRC	DD	DD
B: Forward speed	km/h		1.5	1.5	2	2.5	2.5
C: Straw density	t/ha		1	1	2	3	3

**Table 2:** Independent variables in actual and coded forms and experimental response.

Std	Run	Variable 1 A: furrow openers	Variable 2 B: forward speed	Variable 3 C: straw density	Variable 1 A: Furrow openers (Num)	Variable 2 B: forward speed (km h^-1^)	Variable 3 C: straw density (t ha^-1^)	Response 1 Draft (N)	Response 2 straw cutting efficiency (%)
		Variables in coded form			Variables in actual form				
14	1	0	0	+ α	ITRC	2	3	447.8	51.96
5	2	− 1	− 1	1	SD	1.5	3	289.6	49.18
9	3	− α	0	0	SD	2	2	343.8	54.91
3	4	− 1	1	− 1	SD	2.5	1	369.2	58.2
18	5	0	0	0	ITRC	2	2	391.3	57.17
11	6	0	− α	0	ITRC	1.5	2	332	61.32
16	7	0	0	0	ITRC	2	2	391.3	57.17
17	8	0	0	0	ITRC	2	2	391.3	57.17
4	9	1	1	− 1	DD	2.5	1	432.1	68.44
10	10	+ α	0	0	DD	2	2	383.4	73.87
12	11	0	+ α	0	ITRC	2.5	2	480.5**	54.98
15	12	0	0	0	ITRC	2	2	391.3	57.17
7	13	− 1	1	1	SD	2.5	3	436.2	47.13*
8	14	1	1	1	DD	2.5	3	467.9	62.07
2	15	1	-1	− 1	DD	1.5	1	271.6	81.36**
20	16	0	0	0	ITRC	2	2	391.3	57.17
6	17	1	− 1	1	DD	1.5	3	334.8	75.48
13	18	0	0	− α	ITRC	2	1	373	63.26
1	19	− 1	− 1	− 1	SD	1.5	1	240.2*	56.86
19	20	0	0	0	ITRC	2	2	391.3	57.17

** and * Indicate the highest and lowest values of the responses, respectively.

Table 2, displays the experimental variable factors in both coded and actual forms, along with their corresponding experimental responses. The central composite design (CCD) is a comprehensive arrangement of experimental trials that includes the essential 2^n^ factorial points. This design initiates from the central point and extends to incorporate 2n axial points (where n represents the number of independent variables), in addition to (n_c_) central points used for estimating experimental error. The total number of runs (N) can be determined using Eq. (2).

2 $$N = 2^{n} + 2n + n_{c} = 2^{3} + 2 \times 3 + 6 = 20$$

It was found that a total of 20 experimental runs (N = 20) were necessary for the three levels of independent variables. After selecting the specific variables for the experimental runs, their values were assigned in coded format. The axial points were denoted as ± α, the middle points as 0, and the factorial points as ± 1. To mitigate the impact of uncontrolled factors and errors, the experimental runs were randomized. An empirical model was developed for each response using a second-degree polynomial equation (Eq. 3), incorporating the dependent and independent parameters utilized in the study^41,42^.

3 $$Y = \beta_{0} + \sum\limits_{i = 1}^{n} {\beta_{i} X_{i} } + \sum\limits_{i = 1}^{n} {\beta_{ii} X_{i}^{2} } + \sum\limits_{i = 1}^{n} {\sum\limits_{j = 1 + 1}^{n} {\beta_{ij} X_{i} X_{j} + \varepsilon } }$$

In the experimental context, the anticipated response is symbolized as Y, with the intercept coefficient denoted as α, the linear constant as β, the quadratic constant as γ, and the interaction constant as δ. Within the experiments, 'n' denotes the quantity of factors analyzed and fine-tuned, whereas X_1_ and X_2_ represent the encoded values allocated to the variable factors. The standard error is indicated as ε.

### Optimization of levels of variables

The experimental trial data was analyzed by employing the Central Composite Design (CCD) of Response Surface Methodology (RSM) and utilizing the statistical software Stat-Ease 360 (version 22.0.5), as indicated in Table 2. An Analysis of Variance (ANOVA) was conducted to evaluate the significance of the experimental variables, which include Furrow Openers, Forward Speed, and Straw Density, along with their interactions.

## Results and discussion

The working conditions of the implement had a significant impact on both the force requirements and straw-cutting efficiency of the tool^11,43^. Table 2 indicates that the draft force requirements range from 240.2 N to 480.5 N, while the minimum and maximum straw cutting efficiency are 47.13% and 81.36%, respectively. The ANOVA results for draft force and straw-cutting efficiency are presented in Table 3.

**Table 3:** ANOVA for regression model for draft force requirement and straw cutting efficiency.

Source	Draft force					Straw cutting efficiency					Remark
	SS	df	MS	F-value	p-value Prob > F	SS	df	MS	F-value	p-value Prob > F	
Model	322.65	4	80.66	76.92	< 0.0001**	2036.21	5	407.24	22.09	< 0.0001**	Significant
A-Furrow openers	39.9	1	39.9	38.05	< 0.0081**	1177.53	1	1177.53	63.86	< 0.0001**	Significant
B-Forward speed	174.55	1	174.55	166.46	< 0.0105*	189.46	1	189.46	10.27	0.0064**	Significant
C-Straw density	96.15	1	96.15	91.69	< 0.0213*	343.95	1	343.95	18.65	0.0007**	Significant
AB	12.05	1	12.05	11.5	0.004**	79.25	1	79.25	4.3	0.0571*	Significant
A^2^						246.02	1	246.02	13.34	0.0026**	Significant
Residual	15.73	15	1.05			258.15	14	18.44			
Lack of Fit	15.73	10	1.57	2.45	0.174	258.15	9	28.68	1.44	0.48	Not significant

*, ** Indicate 1% and 5% level of significance, respectively.

The study revealed that the type of furrow opener (A), forward speed (B), and straw density (C) had a significant (P < 0.05) impact on the both draft force requirement and straw-cutting efficiency of furrow openers.

Based on Table 3, the model F-values for draft force and straw cutting efficiency are 76.92 and 22.09, respectively, with a ‘Prob > F’ value of 0.0001, indicating significant models Khuri^44^. The type of furrow opener (A), forward speed (B), and straw density (C) are the major factors that mostly affect draft force, and the interaction effects of furrow opener × forward speed is significant, while the remaining factors were found to be insignificant. In the case of straw-cutting efficiency, the furrow opener type (A), forward speed (B), and straw density (C) had significant effects, along with the interaction effects of A × B and quadratics A^2^, None of the other factors was deemed significant. Table 3 shows that the lack of fit F values for draft force and straw cutting efficiency are 2.45 and 1.44, respectively, which are not statistically significant. The presence of a non-significant lack of fit implies that the model’s F statistics value was significant, indicating a strong influence of the experimental design runs on the independent variables.

**Fig. 5:**
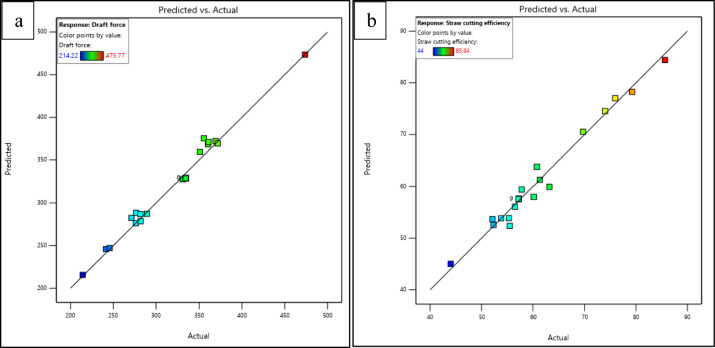
Plot of actual and predicted values of (**a**) draft force and (**b**) straw cutting efficiency.

This also means that there is only a small chance (1.22% and 2.45% for draft force and straw cutting efficiency, respectively) that variations in the results were due to noise. The R^2^ values for the three responses are 0.9835 and 0.9920, indicating that 98.35% and 99.20% of the variation in draft force and straw cutting efficiency, respectively, can be attributed to the experimental variables, while the remaining variation is due to noise. Figure 5, illustrates the actual and predicted values of draft force and straw cutting efficiency obtained from the quadratic model. The graph clearly shows that the actual values of the data points for draft force and straw cutting efficiency obtained through the experimental test runs are nearly adjacent to the predicted values accessed by the model, forming a straight line. This observation suggests that the model exhibits a strong fit and implies that it adequately captures the variations in the independent parameters across the experimental range.

The experimental data were utilized to develop an empirical model through regression analysis. Coded factors were used to predict draft force and straw cutting efficiency based on the variables under study. Equations (4 and 5) depict the final empirical models for draft force and straw-cutting efficiency, respectively.

4 $$\begin{aligned} Draft \;force & = \left( {21.08A + 71.74B + 29.02C + 2.25AB - 2.18AC - 1.23YZ - 43.08A^{2} } \right. \\ & \;\;\; - 0.58B^{2} + 3\left. {.7C^{2} } \right) \left[ {{\text{R}}^{{2}} : \, 0.{98}} \right] \\ \end{aligned}$$

5 $$\begin{aligned} Straw\; cutting\; efficiency & = \left( {9.49A - 3.34} \right.B - 4.23C - 3.20AB + 0.81AB - 0.48BC \\ & \;\;\; + 5.96A^{2} \left. { - 0.28B^{2} - 0.82C^{2} } \right)\left[ {{\text{R}}^{{2}} : \, 0.{99}} \right] \\ \end{aligned}$$

In the equation, A, B, and C represent the coded values assigned to the furrow opener, forward speed, and straw density, respectively. A positive sign within the equation signifies a cumulative impact on the experimental responses, whereas a negative sign indicates a counteractive effect on the responses.

### Combined effect of experimental factors on draft force and straw cutting efficiency

**Fig. 6:**
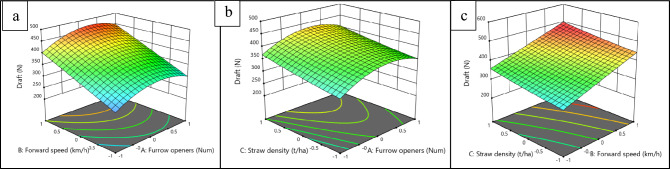
Combined effect of furrow opener, forward speed and straw density on draft force.

**Fig. 7:**
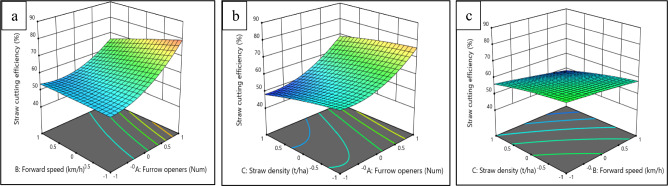
Combined effect of furrow opener, forward speed and straw density on straw cutting efficiency.

To investigate the effects of experimental factors, including the type of furrow opener, forward speed, and straw density, on draft force and straw-cutting efficiency, response surface methodology (RSM) was employed to create three-dimensional response plots. Figures 6, 7, depict these response surface plots, illustrating the interrelationships among the variables. Figure 6, specifically emphases on the combined effect of type of furrow opener (A), forward speed (B), and straw density (C) on draft force.

Within the response surface plot for draft force (Fig. 6), the red area signifies the highest draft requirement, while the blue area represents the minimum draft requirement. In the plot, the furrow openers SD, ITRC, and DD are coded as − 1, 0, and + 1, respectively. From Fig. 6a, it is apparent that regardless of the furrow opener, draft force increases with an increase in forward speed, similar observations were recorded by^11,30^. Notably, the ITRC furrow opener recorded a maximum draft force of 484 N at a forward speed of 2.5 km h^−1^, while the SD furrow opener displayed the lowest draft force of 242 N at a forward speed of 1.5 km h^−1^ (Fig. 6a). The highest draft force is associated with the ITRC furrow opener due to the sliding effect of its inverted T-type design, contrasting with the rolling motion of the disc employed by the SD and DD furrow openers.

The most significant factor influencing draft force was identified to be the interaction effect between forward speed and furrow opener. The reason for the increase in draft force with an increase in operational speed is the higher force required to achieve soil acceleration as the speed increases, as reported by Sahu and Raheman in 2006^26^. Figure 6b, demonstrates that the draft force is notably influenced by the interaction between straw density and furrow opener. Among the furrow openers tested, the ITRC furrow opener showed the highest draft force at a forward speed of 2.5 km h^−1^. Following closely, the DD and SD furrow openers also exhibited increased draft forces at the same speed. These results align with previous findings reported by Badegaonkar^45^. This may be due to the higher cutting force required at higher straw density. The interaction effect of forward speed and straw density is plotted in Fig. 6c. It was observed that both forward speed and straw density significantly influence the draft force. The highest draft force was observed at a forward speed of 2.5 km h^−1^ and a straw density of 3 t ha^−1^, while the lowest draft force was recorded at a forward speed of 1.5 km h^−1^ and a straw density of 1 t ha^−1^. Draft force increases with an increase in both forward speed and straw density. This may be due to the higher force required to achieve soil acceleration as the speed increases and an increase in straw cutting forces as the straw density increases^35^. This study reveals that both forward speed and straw density are directly proportional to the increase in draft force for all three furrow openers, and the ITRC furrow opener requires a higher draft force, followed by the DD and SD furrow openers for the same forward speed and straw density.

The results shown in Fig. 7a, indicate a decrease in straw cutting efficiency with an increase in forward speed, regardless of the furrow opener used. For the DD furrow opener, the highest straw cutting efficiency of 81.36% was recorded at a forward speed of 1.5 km h^−1^, while the SD furrow opener achieved the lowest straw cutting efficiency of 47.13% at a forward speed of 2.5 km h^−1^ (Fig. 7a). The lower efficiency observed at 2.5 km h^−1^ can be attributed to insufficient time for cutting the straw due to the increased forward speed. Among the factors affecting straw cutting efficiency, the interaction effect of straw density and type of furrow opener was found to be the most significant (Fig. 7b). The DD furrow opener demonstrated the highest cutting efficiency, followed by ITRC and SD. The superior straw-cutting efficiency observed in the DD furrow opener is attributed to the result of applying a tensile force on both the straw and the soil^11,14,30^. Additionally, the sticky nature of sandy loam soil in the soil bin led to a reduced occurrence of the hair pinning phenomenon in the DD furrow opener. Previous studies have also reported the superior performance of DD furrow openers in cutting deposited straw by employing a simple shearing and rolling action, with the soil acting as a counter knife^14,30^.

**Fig. 8:**
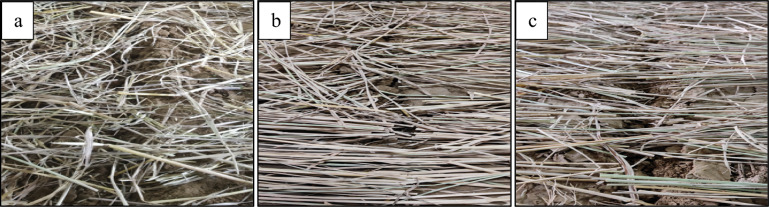
Straw cutting and furrow grooves opening mechanism of (**a**) SD, (**b**) ITRC and (**c**) DD furrow openers.

Similarly, the interaction between forward speed and straw density significantly influenced straw cutting efficiency (Fig. 7c). As both forward speed and straw density increased, the cutting efficiency decreased. This decrease may be attributed to the furrow opener’s limited ability to generate sufficient force for cutting straw at higher densities. Figure 8a–c, provides a visual representation of the variations in cutting performance among the furrow openers (SD, ITRC, and SD).

### Optimization of experimental factors using response surface modelling

The main objective of the experimental study was to determine the favorable conditions for minimizing draft force requirements and maximizing straw cutting efficiency. The optimization of experimental factors was carried out using Stat Ease 360 software. Based on the response surface plot, the optimum conditions were determined. Although there was not a significant difference in draft force requirements between the SD and DD furrow openers, the latter showed better straw cutting performance (Fig. 6). The optimum conditions for minimizing draft force requirements and maximizing straw cutting efficiency are presented in Table 4.

**Table 4:** Optimum conditions for maximizing experimental responses.

Furrow opener	Forward speed (km h^−1^)	Straw density (t ha^−1^)	Draft force requirement (N)	Straw cutting efficiency (%)	Desirability
DD	1.5	1	368.5	74.89	0.94

The minimum draft force requirement and maximum straw cutting efficiency were achieved under the optimal conditions of using a DD furrow opener at a forward speed of 1.5 km h^−1^ and a straw density of 1 t ha^−1^. The desirability of the model, which is close to unity at 0.94, suggests minimal error values and demonstrates the model's suitability for predicting the responses.

Based on the findings of this study, it can be concluded that increased straw-cutting efficiency can be achieved by enhancing the tensile force between the furrow opener and soil medium^14,30,46^. Therefore, when designing and developing new furrow opener and straw-cutting tools for no-till residue conditions, emphasis should be placed on the magnitude of the tensile force applied by the tool on both the soil and the straw. Soil moisture content is another crucial factor affecting the straw cutting ability of furrow openers. The soil surface acts as a cutting board for furrow openers; thus, high soil moisture content reduces straw-cutting efficiency by weakening the soil’s strength and causing the straw to be pushed into the soil without effective cutting^26,47,48^. Similarly, high moisture content in straw also reduces straw cutting efficiency as it becomes easier to press the crop residue into the soil without cutting it^25,38,48^. The forward speed of the furrow opener and straw density are other important parameters that affect straw-cutting efficiency. As the speed of the operation increases, the straw-cutting ability of the furrow openers decreases regardless of the furrow opener type. This can be attributed to insufficient time for cutting the straw due to the increased forward speed. On the other hand, the straw-cutting efficiency decreases with increasing straw density due to the limited ability to generate sufficient force for cutting straw at higher densities. Previous studies have also reported similar findings^11,30,48^. Finally based on the findings of this study, it can be concluded that the DD furrow opener is the most effective tool for cutting straw in conservation tillage.

## Conclusions

The conclusions drawn from the study are based on established findings in the soil bin and aim to highlight practical implications for future research and applications. The draft force requirements and straw cutting efficiency in conservation tillage were significantly influenced by furrow opener type, forward speed, and straw density. Irrespective of the furrow opener, increasing forward speed and straw density increased draft force requirements while decreasing straw cutting efficiency. Among the furrow openers tested, the double disc (DD) exhibited the highest straw cutting ability, while the single disc (SD) required the lowest draft force. In high moisture sandy loam soil, the DD furrow opener demonstrated optimal performance with reduced hair pinning. For no-till straw conditions, it is recommended to use the DD furrow opener to achieve maximum straw cutting efficiency. These findings underscore the importance of selecting appropriate furrow opener based on specific soil and operational conditions to optimize conservation tillage practices.

## Data Availability

The datasets used and/or analysed during the current study are available from the corresponding author on reasonable request.
